# Overexpression of BDNF Suppresses the Epileptiform Activity in Cortical Neurons of Heterozygous Mice with a Transcription Factor Sip1 Deletion

**DOI:** 10.3390/ijms251910537

**Published:** 2024-09-30

**Authors:** Maria V. Turovskaya, Maria S. Gavrish, Viktor S. Tarabykin, Alexei A. Babaev

**Affiliations:** 1 Institute of Neuroscience, Lobachevsky State University of Nizhny Novgorod, 23 Gagarin Ave, 603022 Nizhny Novgorod, Russia; mary_gavrish@mail.ru (M.S.G.); alexisbabaev@list.ru (A.A.B.); 2 Institute of Cell Biophysics of the Russian Academy of Sciences, Federal Research Center “Pushchino Scientific Center for Biological Research of the Russian Academy of Sciences”, Institutskaya st. building 3, 142290 Pushchino, Russia; 3 Institute of Cell Biology and Neurobiology, Charité—Universitätsmedizin Berlin, Charitéplatz 1, 10117 Berlin, Germany

**Keywords:** Sip1, epilepsy, neurons, BDNF, calcium, intracellular signaling, overexpression, kinases, expression

## Abstract

Since genetic mutations during brain development play a significant role in the genesis of epilepsy, and such genetically determined epilepsies are the most difficult to treat, there is a need to study the mechanisms of epilepsy development with deletions of various transcription factors. We utilized heterozygous mice (Sip1^wt/fl^) with a neuronal deletion of the transcription factor Sip1 (Smad interacting protein 1) in the cerebral cortex. These mice are characterized by cognitive impairment and are prone to epilepsy. It is known that the brain-derived neurotrophic factor (BDNF) has a neuroprotective effect in various neurodegenerative diseases. Therefore, we created and applied an adeno-associated construct carrying the BDNF sequence selectively in neurons. Using in vitro and in vivo research models, we were able to identify a key gen, the disruption of whose expression accompanies the deletion of Sip1 and contributes to hyperexcitation of neurons in the cerebral cortex. Overexpression of BDNF in cortical neurons eliminated epileptiform activity in neurons obtained from heterozygous Sip1 mice in a magnesium-free model of epileptiform activity (in vitro). Using PCR analysis, it was possible to identify correlations in the expression profile of genes encoding key proteins responsible for neurotransmission and neuronal survival. The effects of BDNF overexpression on the expression profiles of these genes were also revealed. Using BDNF overexpression in cortical neurons of heterozygous Sip1 mice, it was possible to achieve 100% survival in the pilocarpine model of epilepsy. At the level of gene expression in the cerebral cortex, patterns were established that may be involved in the protection of brain cells from epileptic seizures and the restoration of cognitive functions in mice with Sip1 deletion.

## 1. Introduction

The transcription factor Sip1 is extremely important for the functioning and development of the organism since it is involved in embryonic development regulation, and the disruption of its expression leads to the development of cancerous tumors [1]. Disruption of Sip1 expression in the brain leads to Mowat–Wilson syndrome, characterized by intellectual disability and a tendency to epilepsy [2,3]. Interestingly, complete Sip1 deletion leads to a decrease in the sensitivity of N-methyl-D-aspartate receptors (NMDAR) and α-amino-3-hydroxy-5-methyl-4-isoxazolepropionic acid receptors (AMPAR) of cortical neurons to selective activators [4], a loss of rhythmic Ca^2+^ activity and a decrease in a number of signaling kinases [5]. At the same time, partial Sip1 deletion causes the opposite effect—the sensitivity of NMDAR and AMPAR increases, and the amplitudes of Ca^2+^ signals in response to selective activators increase [4]. A common characteristic of complete and incomplete Sip1 deletion is the disruption of the hypoxic preconditioning mechanism [6] and the possibility of its partial restoration through activation of phosphoinositide 3-kinase [7], the expression of which is not regulated by the Sip1 transcription factor.

At the in vivo level, it has been shown that young (20–30 days) heterozygous mice with a Sip1 mutation in the cerebral cortex, which were administered pilocarpine, exhibit mild signs of epilepsy and survive the experiments [8]. Sip1-deficient mice are characterized by increased anxiety, which is expressed in a decrease in the number of vertical rearings in the open field test. This observation is confirmed in the light-dark test when Sip1-deficient mice prefer to remain in the dark compartment [8,9]. In addition, Sip1-deficient mice were found to have a decrease in motor activity [9], which is also consistent with our data. It is known that high levels of Sip1 expression are shown in serotonergic and dopaminergic neurons, the disruption of which correlates with the development of neurodegenerative processes and probably leads to increased anxiety and reduced motor activity in mice with the Sip1 mutation [10,11,12].

The neuroprotective role of brain-derived neurotrophic factor (BDNF) is well known. Overexpression of BDNF in neurons protects neural networks from damage during ischemia while activating hypoxic preconditioning mechanisms and inhibiting post-hypoxic hyperexcitation in the most vulnerable populations of GABAergic neurons [5,13]. It has been shown that with epileptiform activity, overexpression of BDNF is also able to suppress epileptogenesis through modulation of the GABAergic neurons activity [14,15].

Thus, the aim of this work was to study the molecular mechanisms of epileptogenesis induction in the cerebral cortex of heterozygous mice with a transcription factor Sip1 mutation and the neuroprotective effect of brain-derived neurotrophic factor (BDNF) overexpression.

## 2. Results

### 2.1. The Role of Antioxidants, Anti-Inflammatory Cytokine, Phosphoinositide 3-Kinase Activator and BDNF Overexpression in Suppressing Epileptiform Activity of Cortical Neurons Derived from Heterozygous Sip1 Mice

**Figure 1:**
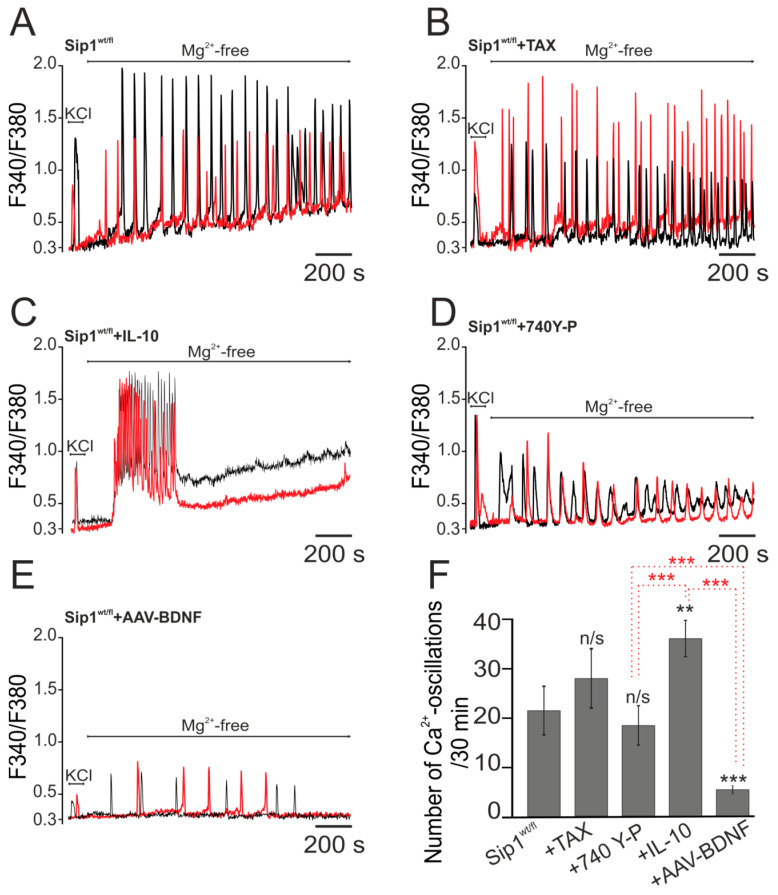
Effects of various compounds with neuroprotective properties and the effect of transduction of the adeno-associated construct carrying the sequence of the brain-derived neurotrophic factor—BDNF on Mg^2+^-free induced Ca^2+^ oscillations in cortical neurons obtained from Sip1^wt/fl^ mice. (**A**) Induction of epileptiform activity in the neuronal network of the cerebral cortex of mice with a deletion of the transcription factor Sip1 using a magnesium-free medium (Mg^2+^-free). (**B**–**D**) Effect of 24 h incubation of Sip1^wt/fl^ neurons with 100 µM antioxidant taxifolin (TAX) (**B**), 1 nM anti-inflammatory cytokine interleukin-10 (IL-10) (**C**) and 1 µM phosphoinositide 3-kinase (PI3K) activator 740Y-P (**D**) on Mg^2+^-induced Ca^2+^-signals. (**E**) Effect of selective BDNF overexpression in Sip1^wt/fl^ neurons on Mg^2+^-induced Ca^2+^-signals. Typical neuronal Ca^2+^ signals are shown. The red and black curves represent the main patterns of neuronal Ca^2+^ signals. (**F**) Average number of Ca^2+^ oscillations in neurons during 30 min of recording with the application of Mg^2+^-free medium as a function of 24 h incubation with cytoprotective agents or with BDNF overexpression. The number of independent cell cultures used for the experiments was 5. Statistical analysis was performed using Student’s *t*-test. ** *p* < 0.01; *** *p* < 0.001; n/s—data not significant (*p* > 0.05, columns without designation).

We have previously shown that complete deletion (homozygous) of the transcription factor Sip1 in cortical neurons leads to disruption of neurotransmission in neuronal networks and, in response to the exclusion of magnesium ions from the extracellular environment, no generation of Ca^2+^ oscillations occurs [7]. In neurons of the cerebral cortex obtained from heterozygous (incomplete deletion) mice, a magnesium-free medium (Mg^2+^-free), as a generally accepted model of epileptogenesis, leads to the opposite effect—activation of high-amplitude high-frequency Ca^2+^ oscillations. In the Sip1^wt/fl^ (heterozygous) neuronal network, in response to Mg^2+^-free, asynchronous Ca^2+^ oscillations are generated (22 ± 12 oscillations during a recording time of 30 min) (Figure 1A,F), occurring with a gradual increase in the basal level of cytosolic calcium ([Ca^2+^]_i_) (Figure 1A). It is known that Ca^2+^ oscillations regulate many physiological processes in the brain [16], including the induction of necrosis and apoptosis. There are studies showing the protective effect of antioxidants in epilepsy [17]. In our experiments, we used the natural antioxidant taxifolin (TAX, 100 µM), which was added to cortical cells from Sip1^wt/fl^ mice for 24 h before induction of epileptiform activity (Figure 1B). It turned out that TAX not only does not affect the generation of Ca^2+^ oscillations and their amplitude but even increases their frequency—28 ± 16 oscillations in 30 min (Figure 1F)—compared to Sip1^wt/fl^ neurons in a magnesium-free medium. Anti-inflammatory cytokine interleukin-10 (IL-10) after 24 h of incubation does not suppress Mg^2+^-free induced Ca^2+^ oscillations and does not significantly suppress their amplitude, but it changes the kinetics of these oscillations (Figure 1C); Ca^2+^ oscillations are characterized by a high degree of synchronization and occur at an elevated baseline [Ca^2+^]_i_ level and spontaneously cease within 7.5 ± 3 min. After incubation of Sip1^wt/fl^ neurons with 1 nM IL-10, a reliable Ca^2+^ oscillations frequency increase occurs (Figure 1F), compared with Sip1^wt/fl^ neurons, but then there was a complete suppression of Ca^2+^ oscillations. IL-10 activates an intracellular signaling pathway involving several signaling pathways, p38, STAT3 and phosphoinositide 3-kinase (PI3K) [18]. Perhaps this is why IL-10 initially led to an increase in Ca^2+^ oscillations and then to their inhibition. Incubation of Sip1^wt/fl^ neurons with a selective activator of PI3K, 1 µM 740 Y-P, leads to significant suppression of the amplitude (Figure 1D) and frequency (18 ± 7 pulses in 30 min) of Ca^2+^ oscillations induced by a magnesium-free medium (Figure 1F), which is probably due to the strict specificity of the activator.

Thus, among the tested cytoprotective compounds, the greatest effect in suppressing epileptiform activity caused by a magnesium-free medium in Sip1^wt/fl^ neurons was demonstrated by the anti-inflammatory cytokine IL-10, which suppressed Ca^2+^ oscillations, and the selective PI3K activator 740Y-P, which reduces the amplitude and frequency of oscillations.

We developed an adeno-associated construct carrying the BDNF sequence. It is known that BDNF overexpression protects brain cells from damage during ischemia and glutamate toxicity [13] and also activates hypoxic preconditioning mechanisms in the population of GABAergic neurons [5]. The key protein activated by BDNF is PI3K, and transduction of this construct into Sip1^wt/fl^ neurons leads to the most pronounced suppression of amplitude (Figure 1E) and frequency (6 ± 3 pulses per 30 min) Ca^2+^-signals (Figure 1F) compared to the neuroprotectors shown above.

Thus, neurons obtained from the cortex of Sip1 heterozygous mice are characterized by increased Ca^2+^ activity upon induction of epileptiform activity using a magnesium-free medium. The antioxidant taxifolin does not affect this process, whereas the anti-inflammatory cytokine IL-10 and the PI3K activator 740Y-P lead to a change in the kinetics of Mg^2+^-free induced Ca^2+^ oscillations, including their suppression or a decrease in their amplitude. On the one hand, IL-10 and 740 Y-P did not affect or even increase the frequency of Ca^2+^ oscillations, but on the other hand, they suppressed the amplitudes of Ca^2+^ oscillations or led to their suppression, which can also be regarded as inhibition of neuronal epileptiform activity in vitro. BDNF overexpression in Sip1^wt/fl^ neurons simultaneously suppresses the frequency of Ca^2+^ oscillations and their amplitudes, which we consider to be the most powerful suppression of Mg^2+^-free-induced epileptiform activity.

### 2.2. BDNF Overexpression Regulates Survival and Neurotransmission Pathways in Cortical Neurons Derived from Sip1^wt/fl^ Mice In Vitro

Based on the neuroimaging results shown above, we decided to investigate the molecular mechanisms of the protective effect of BDNF overexpression during epileptogenesis in vitro. For this purpose, total RNA was isolated from cell cultures obtained from the cerebral cortex of Sip1^wt/fl^ mice on the 10th day of in vitro culture, and the expression of key genes encoding proteins responsible for neurotransmission and neuronal survival during epileptiform activity was analyzed using real-time PCR.

**Figure 2:**
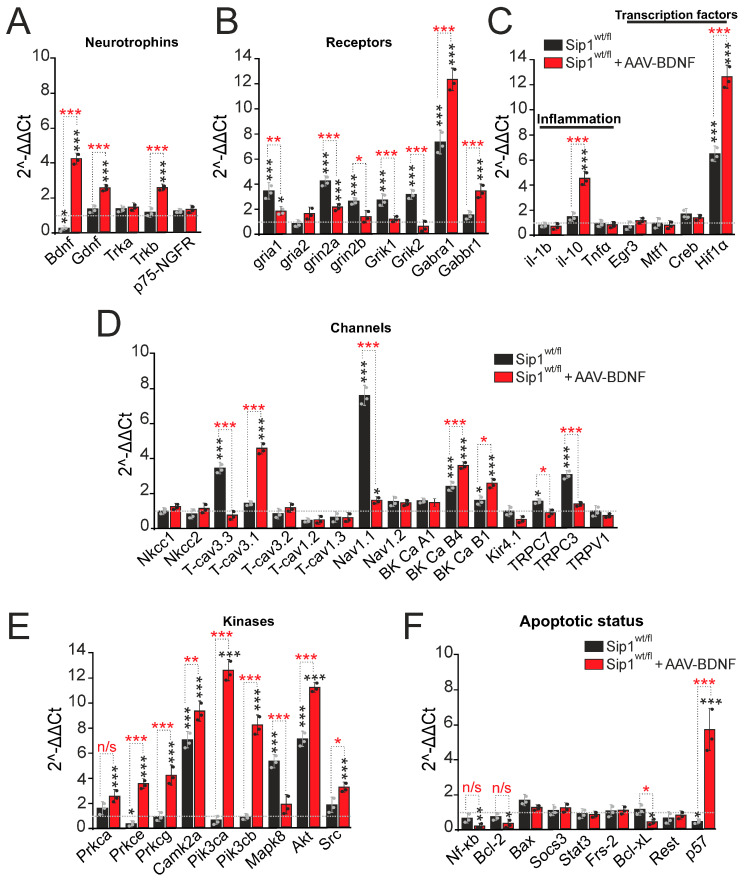
Partial deletion (heterozygosity) of the Sip1 transcription factor in cortical neurons changes the expression level of genes regulating neurotransmission and cell survival signaling pathways. Effect of adeno-associated overexpression of the brain-derived neurotrophic factor BDNF on the expression of the studied genes. (**A**) Expression of genes encoding neurotrophins and their receptors in Sip1^wt/fl^ neurons and Sip1^wt/fl^ neurons transduced with AAV-BDNF. (**B**) Expression of genes encoding subunits of excitatory glutamate and inhibitory (GABA) receptors in Sip1^wt/fl^ neurons without BDNF and with BDNF overexpression. (**C**–**F**) Expression of genes encoding proteins regulating the inflammatory status and transcription factors (**C**), plasma membrane ion channels (**D**), signaling kinases (**E**) and proteins regulating apoptosis (**F**) in Sip1^wt/fl^ neurons without BDNF and with BDNF overexpression. The expression level in cell cultures obtained from wild-type mice is taken as 1 (marked with a dotted line). The results obtained on 3 cell cultures (black and gray circles) are presented. Number of mice for each experimental group = 3. Statistical analyses were performed by Student’s *t*-test. * *p* < 0.05; ** *p* < 0.01; *** *p* < 0.001; n/s—data not significant (*p* > 0.05, columns without designation).

The results of PCR analysis showed that the cortical cells of heterozygous mice with the Sip1^wt/fl^ deletion exhibited a reduced level of BDNF expression (60% of WT cells) compared to cells obtained from cortical cultures of wild-type mice (Figure 2A). In this case, no significant changes in the expression of genes encoding GDNF and receptors for neurotrophins—Trka and Trkb and p75-NGFR are observed (Figure 2A). Overexpression of BDNF in Sip1^wt/fl^+BDNF neurons led to a 4.3-fold increase in the expression of genes encoding BDNF, a 2.6-fold increase in GDNF and Trkb compared to cells from wild-type mice, a 4-fold increase in the expression of genes encoding BDNF, a 2-fold increase in GDNF and a 2.2-fold increase Trkb compared to cells from Sip1^wt/fl^ neurons without AAV-BDNF transduction (Figure 2A).

Changes in the expression and activity of glutamate receptors are involved in the induction of epileptiform activity. In Sip1^wt/fl^ neurons, an increase in the expression of genes encoding the subunits of glutamate receptors GluA1 (AMPAR), NR2A and NR2B (NMDAR), KA1 and KA2 (KAR) was found: gria1 by 3.4 times, grin2a by 4.1 times, grin2b by 2.8 times, and Grik1 and Grik2 by 2.8 and 3.1 times was found compared to the control cells, respectively (Figure 2B). The expression level of the Gabra1 gene, encoding the inhibitory GABA(A) receptor, is 7.2 times higher in Sip1^wt/fl^ neurons compared to the wild type, whereas the expression level of the Gabbr1 gene encoding the GABA(B) receptor does not change significantly (Figure 2B). Overexpression of BDNF leads to a change in the expression level of a number of genes in Sip1^wt/fl^ +BDNF neurons compared with Sip1^wt/fl^ cells without AAV-BDNF transduction (Figure 2B). The expression level of the gria1, grin2a, grin2b, grik1 and grik2 genes decreases by two and more times. The expression level of the Gabra1 and Gabbr1 genes encoding GABA(A) and GABA(B) receptors (Figure 2B, red bars) increases (also almost two times) compared to Sip1^wt/fl^ neurons without BDNF overexpression.

Increased levels of inflammatory cytokines may accompany brain injury during epilepsy. Baseline gene expression levels of the pro-inflammatory cytokine IL-1b and TNFα are not changed in Sip1^wt/fl^ neurons even after AAV-BDNF transduction. Overexpression of BDNF results in an approximately 5-fold increase in the expression level of the gene encoding the anti-inflammatory cytokine IL-10 relative to control and almost three times higher relative to Sip1^wt/fl^ neurons (Figure 2C). Interestingly, expression of the gene encoding the transcription factor Hif1α is approximately 7-fold higher in Sip1^wt/fl^ neurons compared to the wild type (Figure 2C, black bars), approximately 13-fold higher in Sip1^wt/fl^+AAV-BDNF neurons compared to the wild type and 1.8-fold higher than in Sip1^wt/fl^ neurons (Figure 2C, red bars).

Expression of genes encoding some plasma membrane ion channels is higher in cultures obtained from the brain cortex of Sip1^wt/fl^ mice. Deletion of the transcription factor Sip1 leads to an increase in the expression level of the genes T-cav3.3, BK Ca B4, TRPC3 and Nav1.1 encoding T-type calcium channels, large-conductance calcium-activated potassium channels, short transient receptor potential channel 3 and voltage-gated sodium channels by 3.4-fold, 2.8-fold, 3-fold and 7.6-fold, respectively (Figure 2D, black bars). However, upon BDNF overexpression, the expression of the above genes does not occur (except for BK Ca B4), but the level of other genes encoding subunits of plasma membrane channels is changed. Thus, AAV-BDNF transduction into Sip1^wt/fl^ neurons results in a 3.8-fold, 3-fold and 3-fold increase in the expression of T-cav3.1, BK Ca B4 and BK Ca B1, encoding T-type calcium channels and BK channel subunits, compared to wild-type neurons, and significantly higher compared to BDNF-deficient Sip1^wt/fl^ neurons (Figure 2D, red bars).

Deletion of the transcription factor Sip1 results in a 3-fold decrease in the expression level of the Prkce gene, encoding the PKC subunit, whereas the level of the Camk2a, Mapk8 and Akt genes, encoding the kinases CaMKII, mitogen-activated protein kinase 8, and protein kinase B (AKT), increases by 7.2, 5.3 and 7.2 times, compared to the wild type (Figure 2E, black bars). Overexpression of BDNF in Sip1^wt/fl^ neurons resulted in an increase in the expression of genes encoding most of the studied kinases—Prkca, Prkce, Prkcg, Camk2a, Pik3ca, Pik3cb, Akt and Src—by 2.8-fold, 3.7-fold, 4.1-fold, 9.3-fold 12.9-fold, 8.7-fold, 11.9-fold and 3.5-fold, respectively, compared to wild type and with significant difference in comparison to Sip1^wt/fl^ neurons without AAV-BDNF transduction (Figure 2E, red bars). Importantly, the key effect was an increase in the expression level of Pik3ca and Pik3cb genes, encoding the key protective kinase PI3K.

The apoptotic status of Sip1^wt/fl^ neurons is characterized by reduced levels of p57 gene expression in the absence of changes in the expression of other studied genes (Figure 2F, black bars). Overexpression of BDNF in Sip1^wt/fl^ neurons leads to a decrease in the expression of pro-apoptotic Nf-κB and Bcl-xL, but also anti-apoptotic Bcl-2, while the level of p57 expression increases by almost 11.4-fold relative to wild type (Figure 2F, red bars).

Thus, cortical neurons obtained from heterozygous mice with deletion of the transcription factor Sip1 are characterized by a reduced basal expression of genes encoding BDNF, one of the subunits of protein kinase C, as well as a trend towards a decrease in the level of PI3K expression. Such a decrease in gene expression occurs against an increase in the expression of genes encoding several plasma membrane channels, T-type voltage-dependent Ca^2+^ channels, voltage-dependent Na^+^ channels, type 4 BK channels and TRPC3 channels, as well as subunits of excitatory glutamate receptors, NMDAR, KAR and AMPAR. These changes in expression can, in some pathological conditions, determine the increased sensitivity of Sip1^wt/fl^ neurons to damage or hyperexcitation, including epileptiform activity. Positive changes in expression in Sip1^wt/fl^ neurons include an increase in the expression of the gene encoding the GABA(A) receptor, an increase in the number of which can contribute to inhibition in the network during epileptiform activity, and an increase in the level of the Hif1α, Akt and Camk2a genes, which can function as inhibitors of apoptosis.

Overexpression of BDNF in these Sip1^wt/fl^ neurons leads to positive changes in gene expression: there is an increase in the expression of BDNF and its receptor, as well as another neurotrophin, GDNF, compared to both the wild type and Sip1^wt/fl^ neurons without AAV-BDNF transduction. The level of genes encoding NMDAR, AMPAR and KAR subunits decreases compared to Sip1^wt/fl^ neurons and almost corresponds to the expression level in wild-type neurons, which may contribute to a decrease in the Ca^2+^ signals amplitude during epileptiform activity. Also, overexpression of BDNF leads to a reliable increase in genes encoding GABA(A) and GABA(B) receptors, which may contribute to inhibition in the Sip1^wt/fl^ neuronal network during epileptiform activity. Increased baseline expression of the anti-inflammatory cytokine IL-10; transcription factor Hif1α; and genes encoding key kinases PKC, CaMKII, PI3K and Akt (PKB) contribute to the protection of neuronal networks during a number of pathological factors. Among the studied genes encoding cytoplasmic channels, in the case of BDNF overexpression in Sip1^wt/fl^ neurons, there is an increase in the expression of T-type Ca^2+^ channels and type 4 BK channels, which can, on the one hand, contribute to an increase in the Ca^2+^ ions entry into the cytosol during pathologies, but on the other hand, improve neurotransmission and facilitate the signaling in the neuronal network.

### 2.3. Overexpression of BDNF in Cortical Neurons Protects Sip1^wt/fl^ Mice from Death and Epileptiform Seizures Induced by Pilocarpine: Effect on Motor Recovery and Suppression of Hyperexcitability after Epilepsy

**Figure 3:**
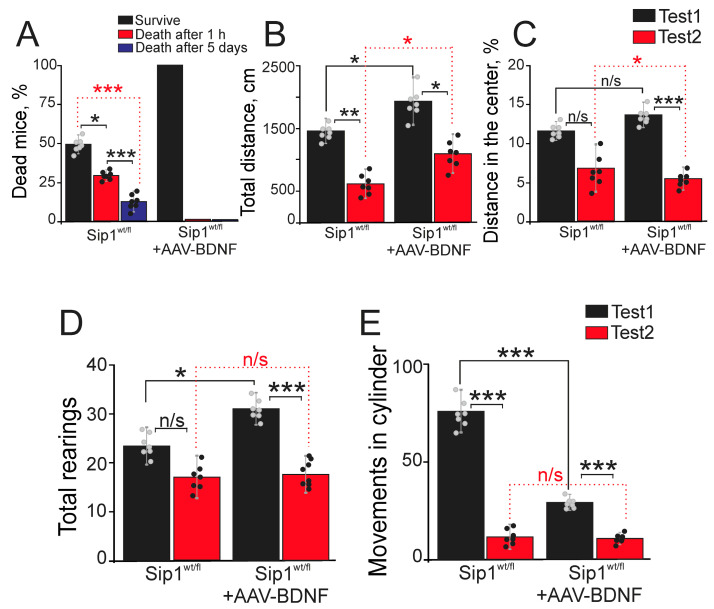
Behavioral tests for motor and cognitive activity of heterozygous Sip1^wt/fl^ mice and Sip1^wt/fl^ mice with selective overexpression of the neurotrophin BDNF in the cortical neurons (AAV-BDNF) before the induction of epileptic seizures (Test 1) and 7 days after pilocarpine injections (Test 2). (**A**) Survival of Sip1^wt/fl^ mice after pilocarpine injection (300 mg/kg) depending on the presence of adeno-associated BDNF overexpression in cortical neurons. (**B**–**D**) Tests for spontaneous activity under novelty conditions (open field test) include the following parameters: total distance traveled (**B**); percentage of distance traveled in the center of the arena (**C**); total motor activity index; total rearings (number of vertical rearings, (**D**)). (**E**) Tests for neurological and motor functions include an indicator—the number of animals turns in a cylinder and is an indicator of anxiety. For each experimental group, 7 mice (black and gray circles) were used. Statistical analyses were performed using two-way ANOVA, followed by Sidak’s multiple comparison test. * *p* < 0.05; ** *p* < 0.01; *** *p* < 0.001; n/s—data not significant (*p* > 0.05).

To study the protective effect of adeno-associated BDNF overexpression on epileptiform activity, we conducted in vivo experiments. The experiments were performed on Sip1^wt/fl^ heterozygous Sip1 mice, which are prone to audiogenic epilepsy and are characterized by behavioral disorders [8,19]. A group of Sip1^wt/fl^ mice without BDNF overexpression was taken as a control group, relative to which the effects of BDNF overexpression in cortical neurons were assessed. After administration of the convulsant, seizures in surviving animals were assessed using the modified Racine scale. In the Sip1^wt/fl^ group without BDNF overexpression, death of mice after convulsant was detected; 27.5 ± 13% died within the first hour after convulsant injection and 12.5 ± 6% on the 5th day (Figure 3A). The surviving 50 ± 12% of Sip1^wt/fl^ mice (Figure 3A—Survive) were assigned seizures status as three points, whereas in those that died after 1 h (Figure 3A—Dead after 1 h) and after 5 days (Figure 3A—Dead after 5 days), seizures status was determined as five points on the Racine scale. Further behavioral tests in the Sip1^wt/fl^ group were carried out among surviving mice with epileptic status three points 7 days after convulsant administration. In the group of Sip1^wt/fl^ mice transduced with AAV-BDNF, pilocarpine injections induced epileptiform activity of three points on the Racine scale in 100% of individuals, all of which survived (Figure 3A) and were used in behavioral tests.

The open field test reflects the spontaneous activity of animals under novel conditions and shows not only general motor activity but also exploratory activity. For Sip1^wt/fl^ mice with BDNF overexpression, a significantly higher total distance traveled was found (1900 ± 399 cm) compared to Sip1^wt/fl^ mice without BDNF overexpression (1500 ± 230 cm) (Figure 3B, Test 1). Seven days after the injection of the convulsant, the mice motor activity in both experimental groups decreased, but with BDNF overexpression, the distance traveled was higher (1099.4 ± 308.9 cm) compared to Sip1^wt/fl^ mice without AAV-BDNF transduction (616.7 ± 253.1) (Figure 3B, Test 2).

Another parameter of the open field test is the percentage of distance traveled in the center (distance in the center), which reflects the anxiety of animals. Without convulsants, no significant differences in this parameter were found between the Sip1^wt/fl^ and Sip1^wt/fl^ + AAV-BDNF groups (Figure 3C, Test 1). After pilocarpine injection, this parameter decreases, which may reflect not only an increase in anxiety but also suppression of general motor activity. However, the percentage of distance traveled in the center tends to decrease significantly in the group of mice with BDNF overexpression, which may indicate a correlation between BDNF expression and an increase in anxiety (Figure 3C, Test 2).

The main indicator of exploratory activity in the open field test is the number of vertical rearings (total rearings), which is higher in Sip1^wt/fl^ mice with BDNF overexpression in the control (before the convulsant injection) (Figure 3D, Test 1), compared to Sip1^wt/fl^ heterozygotes without AAV-BDNF transduction. A repeated series of tests 7 days after the pilocarpine injection leads to a decrease in this indicator in both experimental groups, which does not significantly differ between Sip1^wt/fl^ and Sip1wt/fl + AAV-BDNF (Figure 3D, Test 2). It should also be noted that after the convulsant, the number of urinations and defecations increases in both groups, which can be interpreted as a manifestation of a depressed state and increased anxiety. However, with BDNF overexpression, there is a decrease in defecation and urination during the observation period in the open field test to 3.2 ± 0.8 and 0.0 ± 1.4, respectively, while these indicators in Sip1^wt/fl^ mice without AAV-BDNF transduction are 3.3 ± 1.5 and 1 ± 0.4.

The index of movement in the cylinder before the pilocarpine injection is higher in the Sip1^wt/fl^ heterozygous mice, compared with Sip1^wt/fl^ + AAV-BDNF, which indicates their greater anxiety (Figure 3E, Test 1). However, 7 days after the pilocarpine injection, this index decreases in both experimental groups, and the differences become insignificant between Sip1^wt/fl^ and Sip1^wt/fl^ + AAV-BDNF (Figure 3E, Test 2).

Thus, heterozygous Sip1 mice transduced with AAV-BDNF had higher baseline motor and exploratory activity compared to Sip1^wt/fl^ mice. Pilocarpine injections significantly suppressed motor activity and exploratory activity and increased anxiety in Sip1^wt/fl^ mice with and without BDNF overexpression. However, transduction of AAV-BDNF into the ventricles of the cerebral cortex significantly suppressed neurodegenerative processes after induction of epileptiform activity and correlated with better performance in the open field tests.

The tests for studying neurological and motor functions (sensorimotor examination) included an assessment of the animals’ passage time on flat and round bars of different widths and diameters, respectively. Table 1 shows that, on average, Sip1^wt/fl^ mice with AAV-BDNF transduced into the cerebral cortex pass the tests on an inclined flat bar of different width and a round bar of different diameter faster than heterozygous individuals without BDNF overexpression. But the most interesting results are those showing that 7 days after the pilocarpine injection, Sip1^wt/fl^ + AAV-BDNF mice are many times superior to their relatives without BDNF overexpression in all indices of neurological and motor functions (Table 1).

The startle response assay is used to study many properties of the CNS, including habituation to sound and pre-pulse inhibition PPI (a decrease in the startle amplitude after a preliminary subthreshold stimulus), which reflects the filtering function of sensory inputs to the CNS. A negative PPI value is a sign of this function impairment. A negative PPI value was recorded only in the first test in 37.5% of animals from the Sip1^wt/fl^ group before convulsant injections, while no significant differences in the startle amplitude in response to the sound stimulus were found (Table 2, Mean Max. SR PULSE). At the same time, among Sip1^wt/fl^ mice with BDNF overexpression, no individuals with a negative PPI were found either before or after the convulsant injection (Table 2).

The passive avoidance test is conducted in two stages. In the first stage, the animal is placed in the uncomfortable conditions of a large light chamber with the possibility of moving to a “safe” dark section, and the time to the transition is measured (Latency 1 day). However, the dark section turns out to be unsafe; the animal receives an electric pain stimulus of medium strength there and remembers (or does not remember) that this section is associated with pain. A day later, the animal is placed in the same conditions, and the transition time is measured again (Latency 2 day). If the animal remains in the light section for 180 s, it is considered that it has developed a passive avoidance reflex; if it has moved, it has not. The Delta Latency indicator characterizes the difference between the transition time on the second and first day. Before the injection of convulsants in both experimental groups, the differences in the Delta Latency index were not significant (Table 3, Test 1), whereas 7 days after pilocarpine, the Delta Latency coefficient of Sip1^wt/fl^ mice with BDNF overexpression is higher compared to Sip1^wt/fl^ without AAV-BDNF transduction and is 89.2 ± 21.2, which indicates better learning ability of mice with BDNF overexpression (Table 3). However, a fairly large percentage of animals in both groups did not learn after the administration of pilocarpine, as evidenced by the % of animals entering the dark compartment on the second day (Table 3).

Thus, incomplete deletion of the transcription factor Sip1 in the cortical neurons is accompanied by a number of reliable changes in the behavior of mice. Heterozygous Sip1^wt/fl^ mice are characterized by reduced exploratory activity and learning ability, as well as greater anxiety in a number of behavioral tests. AAV-BDNF transduction and selective overexpression of BDNF in the cortical neurons of Sip1^wt/fl^ mice promotes the activation of their exploratory and motor functions and suppression of anxiety, and thus, such individuals are better adapted to the conditions of a changing environment. Under epileptiform activity induced by pilocarpine injections, 100% of Sip1^wt/fl^ mice with BDNF overexpression survive, whereas more than 50% of Sip1^wt/fl^ mice without AAV-BDNF transduction die within 1 h after pilocarpine injection, which demonstrates a reliable neuroprotective effect of BDNF in epilepsy in vivo. This is confirmed by behavioral experiments 7 days after pilocarpine injections, whereas, in both the control (Sip1^wt/fl^) and experimental (Sip1^wt/fl^ + AAV-BDNF) groups, suppression of almost all studied behavioral functions occurs. However, Sip1^wt/fl^ mice overexpressing BDNF showed significantly better results in behavioral tests and were characterized by better cognitive and motor signs, which may be associated with both the neuroprotective effect of BDNF and its effectiveness in restoring the functions of neural networks in the cerebral cortex in pathologies.

### 2.4. Expression of Key Genes Regulating Neurotransmission and Cell Survival Pathways in the Cerebral Cortex of Sip1^wt/fl^ Mice after Induction of Epileptiform Activity: Protective Role of Selective BDNF Overexpression in Neurons In Vivo

Following epileptiform activity, the neuronal networks of the cerebral cortex undergo morphological and functional rearrangements, which are undoubtedly accompanied by changes in genomic expression. After pilocarpine-induced seizures, surviving Sip1^wt/fl^ mice and Sip1^wt/fl^ mice overexpressing BDNF (Sip1^wt/fl^ + AAV-BDNF) were returned to the SPF vivarium for 7 days, after which behavioral tests (presented above) were performed, immediately after which the animals were euthanized, and total RNA was isolated from their cerebral cortex.

**Figure 4:**
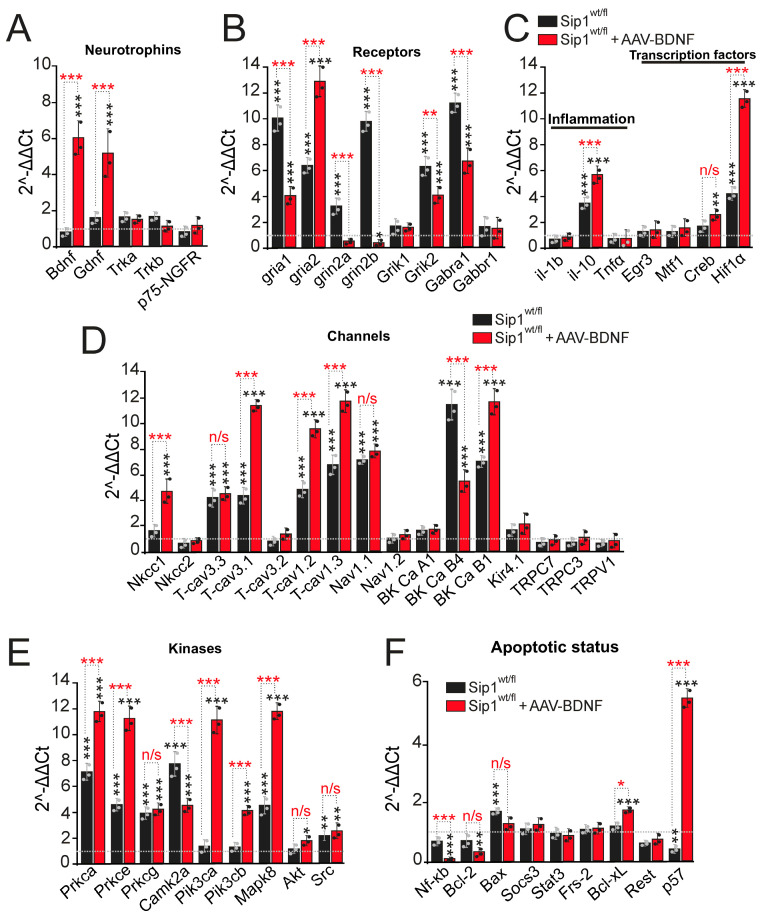
The level of gene expression in the cerebral cortex of Sip1^wt/fl^ mice depending on the presence of adeno-associated BDNF overexpression (Sip1^wt/fl^ + AAV-BDNF) 7 days after epileptiform seizures induced by pilocarpine injections and behavioral tests. (**A**) Expression of genes encoding neurotrophins and their receptors in Sip1^wt/fl^ neurons and in Sip1^wt/fl^ neurons transduced with AAV-BDNF. (**B**) Expression of genes encoding subunits of excitatory glutamate and inhibitory (GABA) receptors in Sip1^wt/fl^ neurons in the absence and presence of BDNF overexpression. (**C**–**F**) Expression of genes encoding proteins regulating the inflammatory status and transcription factors (**C**), plasma membrane ion channels (**D**), signaling kinases (**E**) and proteins regulating apoptosis (**F**) in Sip1^wt/fl^ neurons in the absence and presence of BDNF overexpression. The expression level in the cerebral cortex of Sip1^wt/fl^ mice that died within the first hour after pilocarpine injections is taken as 1 (marked with a dotted line). Number of mice for each experimental group = 3 (black and gray circles). Statistical analyses were performed by Student’s *t*-test. * *p* < 0.05; ** *p* < 0.01; *** *p* < 0.001; n/s—data not significant (*p* > 0.05, columns without designation).

Real-time PCR showed that in the cerebral cortex of Sip1^wt/fl^ mice that survive pilocarpine injections with a score of 3 on the Racine scale, there is no significant increase in neurotrophin expression (Figure 4A, black bars) compared to Sip1^wt/fl^ mice that die within 1 h after pilocarpine injection and are taken as 1 in Figure 4 (control, dotted line). This result is in good agreement with the PCR data obtained on Sip1^wt/fl^ cell cultures in vitro (Figure 2A). Whereas overexpression of BDNF in the cortical neurons of Sip1^wt/fl^ mice leads to an increase in the expression of the gene encoding BDNF and GDNF by 6 and 5 times, respectively, but the level of genes encoding neurotrophin receptors does not change (Figure 4A, red bars).

The expression level of genes encoding subunits of ionotropic glutamate receptors is significantly higher in heterozygous Sip1^wt/fl^ mice that survived after pilocarpine: gria1 and gria2 by 10 and 6.2 times; grin2a and grin2b by 3.2 and 9.8 times; the expression of Grik1 does not change; Grik2 is 6.2 times higher (Figure 4B, black columns). At the same time, these mice are also characterized by an 11-fold increased expression level of the Gabra1 gene encoding the GABA(A) receptor, which may be one of the key values for the survival of these resistant Sip1^wt/fl^ mice during epileptic seizures. Overexpression of BDNF in the cortical neurons of Sip1^wt/fl^ mice is also accompanied by an increased expression level of genes encoding only AMPA receptor subunits, compared to the Sip1^wt/fl^ mice, while the level of other genes encoding NMDAR and KAR subunits is significantly lower, which is a manifestation of the neuroprotective effect of BDNF (which was not observed in in vitro experiments). Thus, in the Sip1^wt/fl^ + AAV-BDNF group, the expression level of the gria1 and gria2 genes is 4 and 12.7 times higher (Figure 4B, red columns). The expression level of the Gabra1 gene is also 6.2 times higher in the cerebral cortex of mice from this group but lower compared to surviving Sip1^wt/fl^ mice (Figure 4B).

Among the genes encoding cytokines and transcription factors, Sip1^wt/fl^ mice showed an increased expression level of the anti-inflammatory cytokine IL-10 by 3.4 times and Hif1α by 4 times (Figure 4C), which correlates with the data obtained on cortical cells in vitro (Figure 2C). A similar effect was found in the group of Sip1^wt/fl^ + AAV-BDNF mice, and the expression level of IL-10 and Hif1α increased by 5.6 and 11.7 times, relative to mice that died within an hour from pilocarpine injections, respectively (Figure 4C, red bars).

Of the 17 genes studied encoding various types and subtypes of plasma membrane ion channels, the expression level of T-cav3.3, T-cav3.1, T-cav1.2, T-cav1.3 (T-type Ca^2+^ channels) increased in the cerebral cortex of Sip1^wt/fl^ mice by 4.5, 4.6, 4.9, and 7 times; Nav1.1 (voltage-dependent Na^+^ channels) by 7.2 times; BK Ca B4 and BK Ca B1 (large-conductance calcium-activated potassium channels) by 11.6 and 7.1 times (Figure 4D, black columns). The expression of the genes encoding the remaining channels did not change significantly. Overexpression of BDNF in cortical neurons of Sip1^wt/fl^ mice resulted in a similar but more pronounced increase in gene expression (except for BK Ca B4) than in the Sip1^wt/fl^ group without BDNF overexpression (Figure 4D, red bars).

Interestingly, the expression level of genes encoding a number of signaling kinases is increased in the cerebral cortex of heterozygous Sip1^wt/fl^ mice epilepsy survivors and Sip1^wt/fl^ + AAV-BDNF mice. In pilocarpine-treated Sip1^wt/fl^ mice, the expression level of Prkca, Prkce, Prkcg, Camk2a, Mapk8 and Src genes increases by 7, 4.6, 3.9, 7.8, and 4.5 times and by 2.2 in the case of Src (Figure 4E, black bars). Overexpression of BDNF in the cortex of Sip1^wt/fl^ leads to a similar increase for Prkcg, Akt, Src аnd more massive increase in Prkca, Prkce, Mapk8 levels, simultaneously with an increase in the expression of PI3K genes (Pik3ca, Pik3cb), respectively (Figure 4E, red bars).

Genes encoding proteins regulating apoptotic status do not significantly change in the Sip1^wt/fl^ cortex of mice surviving after pilocarpine treatment, and there is a significant decrease in the p57 gene by 1.7 fold and an increase in Bax level in 1.5-fold compared to the Sip1^wt/fl^ mice, which dies after pilocarpine (Figure 4F, black columns). BDNF overexpression in the cortex of Sip1^wt/fl^ mice leads to more pronounced effects—suppression of the pro-apoptotic gene Nf-κb and suppression of anti-apoptotic gene Bcl-2—but with a simultaneous increase in pro-apoptotic Bcl-xL almost 2-fold and p57 by 5.4 times (Figure 4F, red columns).

Thus, 7 days after pilocarpine injections, significant changes in the expression of genes encoding primarily the GABA(A) receptor, which promotes inhibition during network hyperexcitation, the anti-inflammatory cytokine IL-10 and the protective transcription factor Hif-1α, as well as protein kinases A, CaMKII, and JNK, occur in the cerebral cortex of surviving Sip1^wt/fl^ mice, which may contribute to the survival of this mice population. Transduction of AAV-BDNF into the cortical neurons of Sip1^wt/fl^ mice leads to a powerful increase in the expression of genes encoding BDNF itself; GDNF; the anti-inflammatory cytokine IL-10; the transcription factor Hif-1α; protein kinases A, CaMKII, JNK; and especially the neuroprotective protein kinase, PI3K, which undoubtedly contributes to the survival of this experimental group. In addition, BDNF overexpression reduces the expression level of genes encoding NMDAR and KAR, which reduces the effect of glutamate toxicity and suppresses the [Ca^2+^]_i_ increase in cortical neurons during epileptiform activity. This occurs against an increase in the expression of genes encoding AMPAR and GABA(A) receptors and a number of key genes encoding plasma membrane channels, which contributes to synaptic plasticity after pilocarpine-induced seizures and network inhibition, as well as modulation of neuronal ion exchange.

## 3. Discussion

Epilepsy is a group of neurological disorders characterized by epileptic seizures, and persistent seizures are a life-threatening condition with a mortality rate of 20% in humans and 40% in rodents [20]. Therefore, there is still an urgent need for further research into the mechanisms of seizure-related mortality. In the present study, we demonstrated the protective effect of BDNF overexpression in protecting the mice brain with a transcription factor Sip1 mutation, which exhibits features of pilocarpine-induced epilepsy and increased Ca^2+^ activity upon induction of epileptiform activity using a magnesium-free medium in an in vitro model.

Mice with the Sip1 mutation are susceptible to CNS developmental disorders, increased nervous system excitability, and are prone to epileptic activity. Using a model of epileptiform activity induction using a magnesium-free medium, we found that the anti-inflammatory cytokine IL-10 exerts a protective effect of suppressing epileptiform activity, which is consistent with the data of other authors, where it was shown that neuroprotective therapy in the epileptic process can lead to less pronounced structural damage, a decrease in epileptogenesis and a milder deterioration in cognitive functions [21]. It is known that in CNS pathology, the level of IL-10 significantly increases in the brain to ensure the survival of nervous tissue and mitigate inflammatory reactions that trigger several pleiotropic signaling pathways [22]. Interleukin-10 at a concentration of 1 ng/mL completely eliminated the development of epileptiform activity in acute rat hippocampal slices under hypoxic conditions. This is associated with the functions of this cytokine as an intercellular mediator of the central nervous system itself and not mediated by the peripheral immune system [23]. These data are consistent with our data when in the culture of Sip1^wt/fl^ neurons under the action of interleukin-10, Ca^2+^ oscillations in a magnesium-free medium attenuate after 7.5 ± 3 min. Stimulation of IL-10 receptors regulates numerous signaling pathways of brain cell survival, including Jak1/Stat3, PI-3-kinase, MAPK, SOCS and NF-kappaB, which ultimately promotes cell survival due to the inhibition of both ligand- and mitochondria-induced apoptotic pathways [24]. Therefore, activation of PI3K by the selective activator 740 Y-P also leads to significant suppression of epileptiform activity induced by a magnesium-free medium.

Unfortunately, many neuroprotective drugs fail to demonstrate efficacy in the treatment of neurodegenerative diseases, as rapid metabolism and poor transport across the blood–brain barrier (BBB) reduce the effect of most compounds [25]. Endogenous BDNF acts, on the one hand, as an important participant in neurogenesis, both in vitro and in vivo [26], but is also of great interest as a tool to prevent neurodegenerative processes in the brain. There is also evidence of the positive effect of exogenous BDNF on brain processes associated with memory, learning, or suppression of excitability [27,28]. It should be taken into account that the administered exogenous BDNF is poorly distributed in the brain, so its concentration at the injection site is usually high and decreases with distance from the injection site [29]. To avoid such effects and to increase BDNF levels in brain tissue at physiological concentrations and with wide distribution, in this study, we used animals that overexpress BDNF, as well as primary neuroglial cultures into which we transduced an adeno-associated construct carrying the brain-derived neurotrophic factor (BDNF) sequence.

It is known that BDNF overexpression protects brain cells from damage during ischemia and glutamate toxicity [13]. Our study showed an excellent neuroprotective effect of BDNF overexpression during the induction of epileptiform activity, which was expressed in a decrease in the frequency and amplitude of neuronal Ca^2+^ signals in response to a magnesium-free medium. Exogenous BDNF activates the release of interleukin-10 and the expression of its mRNA. BDNF can modulate local inflammation in ischemic brain tissues at the level of cells, cytokines and transcription factors [30]. That is, adeno-associated BDNF overexpression potentiates the cytoprotective effect of interleukin-10 during epileptiform activity.

In the present study, we showed that Sip1^wt/fl^ mutant mice had reduced expression of BDNF compared to neurons derived from wild-type mice. The brain-derived neurotrophic factor is a key factor in the induction and maintenance of synaptic plasticity and is also involved in the mechanisms underlying adaptive changes induced by epilepsy [31]. Overexpression of BDNF in Sip1^wt/fl^ neurons leads to increased expression of genes encoding BDNF, GDNF and Trkb compared to cells from Sip1^wt/fl^ neurons without AAV-BDNF transduction, which has a potent neuroprotective effect [32].

When comparing the expression of genes from the cortical neurons from Sip1wt/fl mice with neurons from Sip1wt/fl + AAV-BDNF mice, it was found that the expression of genes encoding the subunits of AMPAR, NMDAR and KAR glutamate receptors was increased in the former group. It is generally accepted that glutamate-mediated hyperexcitation of neurons plays a causal role in the occurrence of seizures. It is well known that agonists of NMDAR or AMPAR receptors can cause seizures in animals or humans, while antagonists of these receptors inhibit seizures in animal models, indicating a potential role for NMDAR and AMPAR receptor antagonists in the development of anticonvulsant drugs [33]. Data from hypoxic models by other investigators demonstrate that KARs, particularly those containing the GluK2 subunit, contribute to alterations in excitatory neurotransmission and seizure susceptibility, particularly during the reoxygenation period in neonatal mice [34,35]. Kainate receptors (KARs) are ionotropic glutamate receptors that mediate rapid excitatory neurotransmission and are also reported to mediate neurotransmission through metabotropic signaling cascades. KARs have been implicated in the pathophysiology of several brain disorders, including epilepsy [36,37,38,39]. Alterations in KAR subunit expression have been reported in both animal models of epilepsy and clinical studies of human temporal lobe epilepsy [34,40,41]. We found in neurons from Sip1^wt/fl^ + AAV-BDNF mice a beneficial effect of increased expression of the gene encoding the GABA(A) receptor, contributing to network inhibition during epileptiform activity. This effect is reasonable since one of the key molecules regulating GABAergic synaptic transmission is brain-derived neurotrophic factor (BDNF) [42]. Our data are consistent with previous studies reporting that BDNF knockdown abolished the effect of preconditioning and promoted the death of GABAergic neurons [5]. Impaired GABAergic transmission due to genetic mutations or the use of GABAergic receptor antagonists causes epileptic seizures, while drugs that enhance GABAergic transmission are used for antiepileptic therapy. In animal models of epilepsy and in tissues of patients with temporal lobe epilepsy, a loss of GABAergic neuron subtypes in the hippocampus is observed. On the other hand, electrophysiological and neurochemical studies indicate a compensatory increase in GABAergic transmission at certain synapses. Also, GABA(A) receptor loss-induced neurodegeneration is accompanied by markedly altered expression of receptor subunits in the dentate gyrus and other hippocampal regions, indicating altered GABA receptor physiology and pharmacology. Such mechanisms may be important for seizure induction, enhancement of endogenous defense mechanisms, and resistance to antiepileptic drug therapy [43].

Expression of genes encoding the pro-inflammatory cytokine IL-10 and the transcription factor Hif1α was increased in neurons from Sip1wt/fl + AAV-BDNF mice compared with Sip1wt/fl without BDNF, while expression of the pro-apoptotic gene Bax was decreased, which together exerted a protective effect [5]. In addition, overexpression of BDNF in cortical neurons from Sip1 mice resulted in increased expression of Bcl-2, Socs3 and Stat3 genes, which may contribute to the protection of neurons from injury in epilepsy. Previous studies have shown that SOCS3 expression can be induced by the JAK/STAT signaling pathway, especially STAT3 [44,45], indicating that SOCS3 is a STAT3-inducible gene. Specifically, phosphorylated STAT3 requires dimerization and translocation to the nucleus, where it induces transcription of SOCS3 genes. Expression of SOCS3 in neurons has been shown to promote excitotoxic neuronal death in vitro. The study by these authors demonstrated the pattern of SOCS3 expression in neurons and its negative regulatory effect on the expression of anti-apoptotic protein, which consequently promotes neuronal death after complete spinal cord injury in adult rats [46]. Also, expression of anti-apoptotic genes Stat3, Socs3 and Bcl-xL significantly increases 24 h after hypoxia episodes in BDNF-transduced cultures compared to controls. In turn, expression of pro-apoptotic (Bax, Casp-3 and Fas) and pro-inflammatory (IL-1β and TNFα) genes decreases after hypoxia episodes in cultures with BDNF overexpression [5].

We attempted to show which of the genes involved in epileptogenesis were analyzed based on the expression of a number of genes involved in neuroplasticity. We analyzed key genes encoding proteins responsible for neurotransmission and neuronal survival during pilocarpine-induced epileptiform activity in an in vivo model. The results of the in vivo study are in good agreement with the data we obtained in cellular models. Depending on the pilocarpine administration protocol and the rodent species, mortality in rodent models can reach 40% [47]. Induction of epileptiform seizures induced by pilocarpine injections provoked an increase in the expression level of genes encoding BDNF and GDNF in Sip1^wt/fl^ mice and Sip1^wt/fl^ + AAV-BDNF mice, but the level of genes encoding neurotrophin receptors did not change. Epilepsy conditions lead to an increase in the expression of genes encoding AMPAR and NMDAR subunits and an increase in the expression of genes encoding GABA(A) receptors, which may contribute to inhibition in the Sip1^wt/fl^ neuronal network during epileptiform activity. Overexpression of BDNF in neurons from Sip1^wt/fl^ mice leads to an increase in the expression of genes encoding AMPAR, the Grik2 subunit of KAR and the GABRA1 subunit of GABAR. We noticed that IL-10 gene expression in neurons from Sip1^wt/fl^ mice is approximately two times lower than in neurons from Sip1^wt/fl^ mice with BDNF overexpression. After induction of epileptiform activity, a tendency towards an increase in interleukin-10 gene expression is observed in both groups of studied neurons on day 7, but the ratio of IL-10 expression levels in these groups remains the same before and after epileptiform activity. Consistent with our data are the results of Youn et al., who found that IL-10 expression is not observed immediately after neuronal exposure but takes 2 to 3 days to be detectable in plasma in neonatal seizures induced by hypoxic-ischemic encephalopathy [48]. In the latent phase, IL-10 showed significant upregulation with higher expression at 21 days post-epileptic compared to the 7-day group. The rapid and marked expression of IL-10 in the latent phase may also correspond to changes in the expression of genes involved in the inflammatory response that occurs during the latest phase [49,50].

An experimental model of pilocarpine seizures stimulated the expression of the HIF-1α gene (2-fold increase) in neurons from Sip1^wt/fl^ mice overexpressing BDNF. The study by Li Jie suggests that the activation of HIF-1α may be involved in the process of epileptogenesis but not in the acute stage of epilepsy. Modulation of HIF-1α may offer a new therapeutic target in epilepsy [51]. We found that after the induction of pilocarpine seizures in neurons from Sip1^wt/fl^ + AAV-BDNF mice, there was a strong increase in the expression of T-cav3.1, T-cav1.2, T-cav1.3, Nav1.6 and BK channels. T-type calcium channels play a role in the burst excitation of neurons and are involved in several seizure models. The role of the T-type calcium channel Cav3.1 (α1G) in a model of temporal lobe epilepsy induced by kainic acid (KA) has been described in the literature. It has been shown that the T-type calcium channel α1G plays a modulatory role in the duration and frequency of seizures in the hippocampus, as well as the epileptogenicity of KA-induced temporal lobe epilepsy in mice, mainly during acute periods [52]. There is also evidence of an increase in the number of neuronal L-type Cav 1.3 calcium channels in various animal models of epilepsy [53]. L-type Ca^2+^ channels open readily during membrane depolarization and allow Ca^2+^ entry to the neurons. Thus, L-type Ca^2+^ channels regulate cell excitability and trigger various Ca^2+^-dependent physiological processes such as excitation–contraction coupling in muscle cells; gene expression; synaptic plasticity; neuronal differentiation; hormone secretion; and pacemaker activity in the heart, neurons and endocrine cells [54]. The sodium channel subtypes Nav 1.2 and Nav 1.6 are the two predominant forms in excitatory cortical pyramidal neurons with localization specificity and can interact to initiate and propagate action potentials [55]. With our scientific data, we understand that increased expression of Nav 1.2 channels promotes neuronal excitability, while the decreased expression we observed after seizure induction has the opposite effect. In this work, we have shown changes in the expression of ion channels that direct neurons toward the development of epilepsy. Excitation- and calcium-activated potassium (BK) channels promote early spiking in neurons, and studies by other authors also show that BK channels play a pathological role in increasing excitability at seizure onset. Here, we investigated changes in BK channel expression in three subunits: A1, β4 and B1. Seven days after pilocarpine-induced seizures, we found that the predominant effect was an increase in the expression of the β4 subunit in both Sip1^wt/fl^ mice and Sip1^wt/fl^ + AAV-BDNF mice compared to the expression of this subunit before seizure activation. Most likely, this is a protective mechanism for the resistance of neurons to damage after epileptiform activity. In support of our results, a group of American scientists, using heterozygous β4-subunit knockout mice, found that reduced expression was sufficient to increase the sensitivity of neurons to epileptic seizures [56].

Expression of genes encoding TRPC3 and TRPC7 channels decreases in both experimental groups of mice 7 days after induction of pilocarpine seizures, which has a positive effect on neuronal survival, although, before the experiments, the expression rates of these genes were above unity in neurons without BDNF overexpression. Accumulated data indicate that TRPC channels play a critical role in various aspects of epileptogenesis. TRPC1/4 channels make a major contribution to the occurrence of non-synaptic epileptiform bursts in CA1 and the lateral septum. TRPC7 channels play a critical role in the synaptic activation of epileptiform bursts. A decrease in spontaneous and epileptiform bursts in CA3 correlates with a decrease in pilocarpine-induced epileptiform bursts in vivo in TRPC7 knockout mice. TRPC channels also significantly contribute to epileptiform burst-induced neuronal death [57]. TRPC3 channels are known to be effectors of the brain-derived neurotrophic factor (BDNF)/trkB signaling pathway. Given the long-postulated role of BDNF in epileptogenesis, TRPC3 channels may be a critical component underlying the pathophysiology of seizures and epilepsy [58]. In our study, we attempted to demonstrate the role of TRPC3 channels in pilocarpine-induced status epilepticus. We found that downregulation of TRPC3 channel-encoding genes reduced behavioral manifestations of seizures, indicating a significant contribution of TRPC3 channels to pilocarpine-induced status epilepticus. Our results demonstrate that BDNF overexpression in Sip1 knockout mice uniquely reduces pilocarpine-induced epileptiform activity through TRPC3 channel expression.

We found interesting changes in the expression of genes encoding kinases in Sip1^wt/fl^ mice and Sip1^wt/fl^ + AAV-BDNF mice before and after induction of pilocarpine seizures. The initial level of Prkca, Camk2a, Mapk8 and Akt gene expression in neurons from Sip1^wt/fl^ mice was increased relative to the control (wild-type mice) by three or more times. After 7 days after pilocarpine seizures, in the surviving mice of this group, we observed a multiple increase in the Prkca and Prkce, Camk2a and Mapk8 genes expression thirty or more times exceeding the control. In addition, we found an increased expression of the genes encoding Prkce and two PI3K subunits, and in almost all cases, the expression level of these genes was several times higher relative to neurons from Sip1^wt/fl^ mice. On the contrary, the expression of Akt significantly decreased relative to the control on the seventh day after the induction of pilocarpine seizures. The PI3K/Akt signaling pathway is a classical signaling pathway with biological effects. It plays multiple roles in regulating cell growth, proliferation, differentiation and survival. The PI3K/Akt signaling pathway is widely distributed in the nervous system, which may promote neuronal survival through the regulation and control of apoptosis and autophagy. Akt activation is the key to its anti-apoptotic effect. Akt is a direct substrate of PI3K. The expression of p-Akt may indicate the activation of the PI3K/Akt pathway. The role of the PI3K/Akt pathway in promoting cell survival also has a number of important biological effects through the regulation of downstream proteins associated with apoptosis. Among them, the regulation of the anti-apoptotic protein Bcl-2 and the pro-apoptotic protein Bad in the Bcl-2 family plays a significant role in diseases of the nervous system [59].

There are many studies devoted to the methods of protecting the brain from epilepsy induced by various convulsants. It has been shown that the use of viral constructs AAV-Syn-BDNF-eGFP and AAV-Syn-GDNF-eGFP and, accordingly, overexpression of BDNF and GDNF in C57Bl6 mice has a multidirectional effect on the weight and body length characteristics of mice in the early postnatal period; however, it ensures the animal’s resistance to the development of seizure activity under audiogenic stimulation in the late postnatal period and preserves basic behavioral reactions, emotional status and cognitive abilities of mice after simulated stress [15].That is, it is known that the use of viral constructs does not cause cognitive impairment in wild-type mice. In our experiments, AAV-Syn-BDNF-eGFP Sip1^wt/fl^ mice were used, which are characterized by cognitive and motor impairments prone to epilepsy, and BDNF overexpression not only did not worsen their condition but, on the contrary, completely suppressed the death of mice upon pilocarpine injections and contributed to the restoration of cognitive functions. Antiepileptic effects of BDNF-secreting ARPE-19 cell lines implanted in the hippocampus were shown in Sprague-Dawley rats. Recognition memory was assessed using the novel object recognition (NOR) test. All rats spent more time studying the novel object in the initial phase before the epileptogenic stroke (pilocarpine model). The epileptic state is associated with memory impairment in this test in rats, but BDNF administration correlated with a more intense study of the novel object by rats after epilepsy. Thus, BDNF treatment significantly improved memory function in rats after pilocarpine-induced epilepsy [60]. Our results not only show an improvement in cognitive functions of Sip1^wt/fl^ mice after epilepsy but also in a number of behavioral tests; BDNF overexpression contributed to the improvement of these parameters in Sip1^wt/fl^ mice before pilocarpine injections, which indicates the ability of BDNF to improve the neurological status of mice with genetic defects of the cerebral cortex.

We conclude that seizure-induced changes in the expression of various channel subtypes, protein kinases and transcription factors represent a mechanism that alters neuronal excitability. Thus, all channel subtypes described by us exhibit plasticity in response to the excitatory action of pilocarpine in the cortex of Sip1-deleted mice, and BDNF overexpression is able to shift gene expression patterns toward suppression of epileptogenesis.

## 4. Materials and Methods

Experimental protocols were approved by the Bioethics Committee of the Institute of Cell Biophysics. Experiments were approved by the Bioethics Committee of Lobachevsky State University of Nizhny Novgorod (protocol No. 82 dated 15 May 2024). Experiments were carried out according to Act708n (23 August 2010) of the Russian Federation National Ministry of Public Health, which states the rules of laboratory practice for the care and use of laboratory animals, and the Council Directive 2010/63 EU of the European Parliament on the protection of animals used for scientific purposes.

### 4.1. Animals and Experimental Strategy

We used Sip1 mutants, obtained in Higashi laboratory [61]. In mice of this line, the seventh exon of Sip1 is flanked by the loxP sites (Sip1^fl/fl^) necessary for the Cre recombinase. When crossing Sip1^fl/f l^ * Nex^+/+^ mice with Sip1^+/+^ * Nex^Cre/Cre^ mice [62], which synthesize Cre recombinase only in post-mitotic cells of their neocortex, a conditioned mutant for the Sip1 gene (knockout) is obtained. The simultaneous presence of the Sip1 gene with the loxP sites and Cre recombinase in post-mitotic cells of the cerebral cortex leads to a partial deletion of Sip1 and, as a consequence, translation of damaged protein product of Sip1 with loss of its function. Mice were kept in SPF cages 40 × 25 × 15 cm under standard laboratory conditions: a 14 h light circuit, 22 °C. Animals had free access to food and water.

For genotyping, the tail cuts were dissolved in 0.3 mL of lysis buffer (100 mM Tris-HCl pH 8.5, 5 mM EDTA, 200 mM NaCl, 0.2% SDS, 100 μg/mL proteinase K) at 55 °C for 2–10 h. The non-lysed tissue was removed by centrifuging the samples for 5 min at 13,000 rpm. The DNA was precipitated by adding an equal volume of isopropanol, then mixed and centrifuged (15 min, 13,000 rpm). The precipitated DNA was washed twice in 80% ethanol, air-dried and dissolved in 50 μL of sterile distilled water. All PCR reactions were carried out in a volume of 20 μL.

The following primers were used to determine the amplified product. Sip1-floxed allele and wild-type allele: 5′-TGGACAGGAACTTGCATATGCT-3′; 5′-GTGGACTCTACATTCTAGATGC-3′. Amplification program was as follows: 95 °C, 10 s; 59 °C, 20 s; 72 °C, 40 s; 34 cycles. The wild-type allele product is ~450 bp, Sip1-floxed ~600 bp. Primers for amplification of the NexCre allele and the wild type allele: 5′-CCGCATAACCAGTGAAACAG-3′; 5′-GAGTCCTGGAATCAGTCTTTTCT-3’; 5′-AGAATGTGGAGTAGGGTGAC-3′. Amplification program: 95 °C, 20 s; 54 °C, 30 s; 72 °C, 60 s; 40 cycles. The product of the wild-type allele is ~750 bp, and the NexCre allele is ~500 bp.

Mice were kept in SPF cages 40 × 25 × 15 cm under standard laboratory conditions: a 12 h light circuit, 22 °C. Animals had free access to food and water. For housing, individually ventilated GM500 cages manufactured by Tecniplast (Italy) with a floor area of 501 cm^2^ were used. In the nests, the harem type of housing (2 females + 1 male) was carried out. After weaning, on the twenty-first–thirtieth postnatal day, the offspring were transplanted into a separate cage, and the material for genotyping (tip of the tail) was taken. All animals were housed with littermates of the same sex in a group of 2–5 mice.

**Figure 5:**
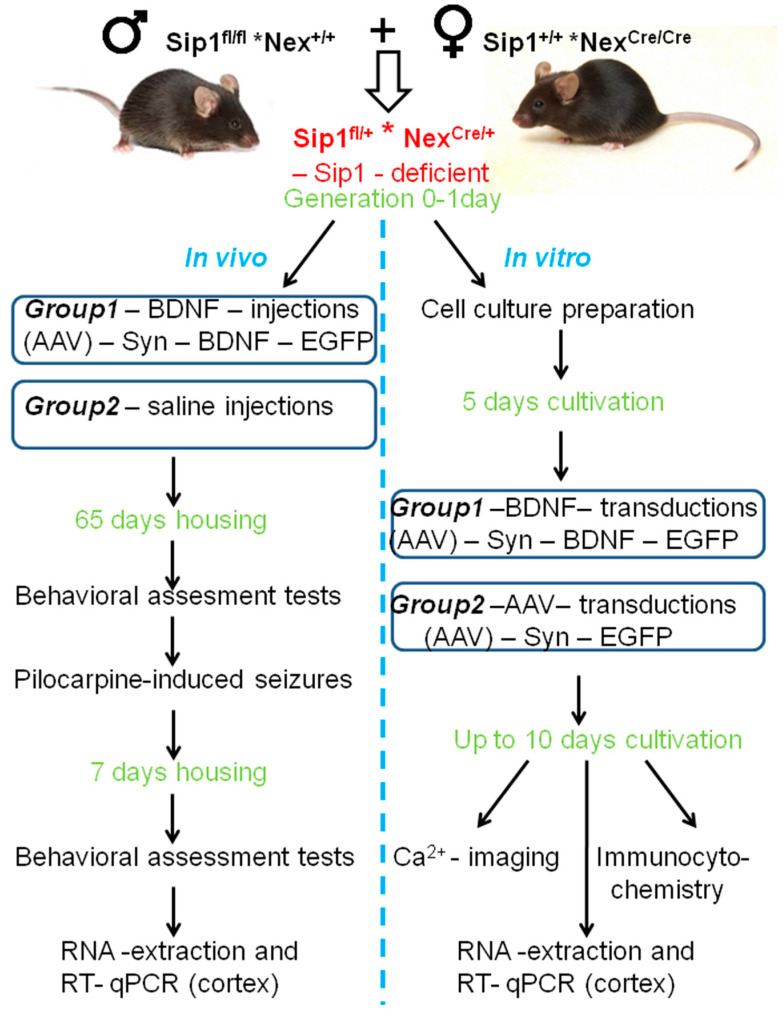
An experimental strategy. In the generation, there were Sip1-deficient (Sip1^fl/+^ * Nex^Cre/+^) mice. The cerebral cortex from one mouse was used to obtain the cell culture. The cell suspension was inoculated at the rate of one cortex per 10 Petri dishes. The cells were cultured for up to 10 days in vitro (DIV). Then, four Petri dishes were used for [Ca^2+^]_i_ dynamics registration, total RNA was isolated from the other four Petri dishes, and the RT-qPCR was performed. Two Petri dishes were used for immunocytochemistry. This approach makes it possible to reliably compare neuroimaging data with changes in gene expression. Simultaneously with the data analysis, mice were genotyped, and then the results were divided into groups. For in vivo experiments, after birth, Sip1-deficient mice were divided into two groups: the first group underwent intracerebroventricular injection with the viral vector (AAV)-Syn-BDNF-eGFP; the second group received saline solution. The mice were then returned to the parental nest and raised for 65 days, after which behavioral tests were performed, and activity patterns were determined. After this, pilocarpine-induced seizures were modeled, and status epilepticus in mice was determined. Total RNA was isolated from the cerebral cortex of deceased mice for further qPCR analysis. Surviving mice were returned to the SPF vivarium for 7 days and kept under standard conditions. Then, behavioral tests were repeated with these Sip1-deficient mice. Afterward, the animals were sacrificed by cervical dislocation, and total RNA was isolated from the cerebral cortex for qPCR analysis.

Newborn mice were used to obtain neuroglial cell cultures of the cerebral cortex in vitro and to achieve BDNF overexpression in vivo using intracerebroventricular viral infection with adeno-associated vector (AAV)-Syn-BDNF-eGFP. The tails of these mice were frozen and stored for subsequent genotyping. An experimental strategy is shown in Figure 5. Briefly, cells were grown up to 10 days in vitro (DIV), loaded with the Fura-2 calcium-sensitive probe, and [Ca^2+^]_i_ dynamics were registered. At the data analysis stage, genotyping was carried out, and the distribution of experimental data was in accordance with the genotype of animals, while at the previous stages, blinded experiments were carried out.

### 4.2. Induction of BDNF Overexpression in the Cortical Neurons In Vivo

Neonatal Sip1-deficient mice were given an intracerebroventricular injection of adeno-associated viral vector (AAV)-Syn-BDNF-eGFP carrying the BDNF sequence. Briefly, the neonatal mouse (pup) was transferred from a warm environment onto a cold metal plate to induce hypothermia anesthesia. After 2–3 min, the mouse ceased motor activity, indicating the effectiveness of anesthesia. Intracerebroventricular injection of AAV into neonatal mice was performed using a 10 µL injection syringe with a 30G needle filled with 5 µL of diluted AAV with 0.05% trypan blue. The injection site is located approximately 0.8–1 mm lateral from the sagittal suture, halfway between lambda and bregma. These landmarks are visible through the skin at P0. After the injection of both hemispheres, the mouse was placed back on the warming pad until its body temperature and skin color returned to normal, and the pup began to move. The mouse pup was then returned to its biological mother. The injection procedure was repeated with other mice in the generation. Sip1-deficient mice from the same generation, which were injected with saline instead of (AAV)-Syn-BDNF-eGFP into the ventricles of their brain hemispheres, served as a control. The animals from both groups were then kept under standard conditions for 65 days.

### 4.3. Cell Culture Preparation

Mixed neuroglial cell cultures were prepared as described in detail previously [13,63,64,65]. The cortex of one mouse was used to obtain ten Petri dishes with culture to avoid the variation in the gene expression and signaling system activity between individual mice. Briefly, 0–1 day-old pups were euthanized by halothane overdose and decapitated. The mouse cerebellar cortex was excised with clippers, placed in a test tube and incubated for 2 min; the supernatant was removed with a pipette. The cells were then covered with 2 mL trypsin (0.1% in Ca^2+^- and Mg^2+^-free Versene solution, SAFC, Cat. #59427C) and incubated for 15 min at 37 °C under constant shaking at 600 rpm. Trypsin was then inactivated by an equal volume of cold embryo serum, and the preparation was centrifuged at 300 g for 5 min. The supernatant was discarded and cells were washed twice with Neurobasal A medium (Thermo Fisher Scientific, Waltham, MA, USA, Cat. #10888022) before being resuspended in Neurobasal-А medium containing glutamine (0.5 mM, Sigma-Aldrich, St. Louis, MO, USA, Cat. #G7513), B-27 (2%, Thermo Fisher Scientific, Waltham, MA, USA, RRID: CVCL_A315) and gentamicin (20 μg/mL, Sigma-Aldrich, St. Louis, MO, USA, Cat. #G1397). An amount of 200 μL of the suspension was put in a glass ring (internal diameter of 6 mm) resting on a round 25 mm coverslip (VWR International, Radnor, PA, USA, Cat. #48382-085) that had been coated with poly-L-lysine. The glass ring was removed after a 5 h incubation period in a CO_2_ incubator (37 °C), and the culture medium (2/3 of the volume) was replaced every 3 days. The density of plated cells was 15,000 cells/sq·cm, and the age of the neuronal cell culture was 10 days in vitro (DIV).

### 4.4. Induction of BDNF Overexpression in Neurons In Vitro

Mixed neuroglial cortical cell cultures were transduced with (AAV)-Syn-BDNF-eGFP virus vector to induce BDNF overexpression in neurons. The structure of this adeno-associated viral construct was described previously [13]. To reach selective BDNF overexpression in neurons, the sequence of human synapsin (hSyn) promoter was incorporated in the vector. (AAV)-Syn-BDNF-eGFP construct was added to the cultures at 5 DIV (construct dilution 1:125). BDNF overexpression was observed mainly in cortical neurons 24 h after the transduction and maintained until 10 DIV.

### 4.5. Fluorescent Ca^2+^ Measurements

Experiments were carried out in the daytime. The measurements of [Ca^2+^]_i_ were performed by fluorescence microscopy using Fura-2/AM (Thermo Fisher Scientific, Cat. #F1221), a ratiometric fluorescence calcium indicator. Neurons were loaded with the probe dissolved in Hanks balanced salt solution (HBSS) composed of (mM): 156 NaCl, 3 KCl, 2MgSO_4_, 1.25 KH_2_PO_4_, 2CaCl_2_, 10 glucose and 10 HEPES, pH 7.4, at a final concentration of 5 μM at 37 °C for 40 min with subsequent 15 min washout. A coverslip containing the cells loaded with Fura-2 was then mounted in the experimental chamber. To measure cytosolic Ca^2+^ concentration, we used the Carl Zeiss Cell Observer, (Oberkoche, Germany) and an inverted motorized microscope Axiovert 200M (Carl Zeiss, Oberkoche, Germany) with a high-speed monochrome CCD-camera AxioCam HSm with a high-speed light filter replacing system, Ludl МАС5000. Fura-2 excitation and registration were recorded using a 21HE filter set (Carl Zeiss, Oberkoche, Germany) with excitation filters BP340/30 and BP387/15, beam splitter FT-409 and emission filter BP510/90, objective lens Plan-Neo fluar 10×/0.3, excitation light source HBO 103W/2. Calcium responses were shown as a ratio of fluorescence intensities of Fura-2 excitation at 340 and 380 nm. To discriminate neurons and astrocytes, a short-term application of 35 mM KCl at the end of experiments was used. This method was described in detail in our previous work [66,67]. Therefore, we determined the frequencies and amplitudes of Ca^2+^ oscillations in the model of epileptiform neuronal network activity as the number of Ca^2+^ spikes during recording and as (Δ)—Fmax–Fmin of Fura-2 fluorescence, respectively. ImageJ 2002 software (developed by LOCI at the University of Wisconsin, Madison, WI, USA, available at https://imagej.nih.gov/ij/download.html, accessed on 18 May 2023, RRID: SCR_003070) was used to analyze data.

### 4.6. Induction of Epileptiform Activity In Vitro

For the induction of epileptiform activity in the neuronal network, we used a generally accepted model. In the Mg^2+^-free model, Mg^2+^ ions were excluded from the HBSS medium [7] and added to the imaging chamber using a perfusion system. In the case of a Mg^2+^-free medium, MgSO_4_ in HBSS was replaced by an osmotically equivalent concentration of Na_2_SO_4_.

### 4.7. Pilocarpine-Induced Seizures

To simulate seizures, animals were administered methylscopolamine nitrate (Sigma) in 0.9% NaCl at a concentration of 1 μg/kg subcutaneously to reduce the peripheral effects of pilocarpine, and then, after 30 min, an intraparenteral injection of the convulsant pilocarpine hydrochloride (Sigma) in 0.9% NaCl at a concentration of 300 mg/kg was performed. The seizures and behaviors were recorded continuously for more than 120 min with video monitoring after drug injection. Seizures were scored based on behavior findings using a modified Racine scale (Table 4).

### 4.8. Behavioral Assessment Tests

A series of behavioral tests were performed twice: when the animals reached 65 days of age and 7 days after pilocarpine-induced seizures. The tests were performed in the following sequence each day at the same time between 10:00 and 13:00 p.m. One hour before the test, the animals were placed in the home cage in the test room for adaptation.

#### 4.8.1. Sensorimotor Tests

The test evaluates the motor functions and some reflexes. To test the hind limb extension reflex, the mouse was suspended by the tail for 1 min. The normal manifestation of this reflex implies an extension of hind limbs; contracting one limb towards the midline of the abdomen or clasping the limbs is considered abnormal [69]. To detect a deficiency in the motor coordination and balance, a set of sensorimotor tests was used, such as the balance beam walking test. During the experiment, the time taken by the animal to perform each of the assigned tasks was recorded. The maximal time of observation was 120 s. The mice were tested according to the following criteria:

(1) Walk on a horizontal beam. Flat beams 1, 2 and 3 cm wide or 3 and 0.5 cm round beams were used. The mouse was placed in the middle of a 50 cm long horizontal beam, with its ends fixed on two platforms 50 cm above a soft base. The time taken by the mouse to reach one of the platforms was measured. If the animal slipped off the beam, the result was considered to be 120 s.

(2) The hanging wire test. The mouse was suspended by their forelimbs on a horizontal wire hanging between two platforms at a height of 50 cm above a soft base. The time before the mouse fell was measured.

(3) Turning around on an inclined screen. The mouse was placed on an inclined screen to face down the slope (a 20 × 20 cm wire mesh platform fixed at an angle of 45°) at a height of 50 cm above the table. The time before the mouse turned around to face up the slope was measured.

(4) Turning around in a cylinder. The mouse was placed into a closed cylinder (3 cm in diameter and 13 cm long) facing the cylinder wall. The time before the animal turned around to face the opposite direction was measured.

#### 4.8.2. The Open Field Test

To study the motor and exploration activity of mice placed in an unfamiliar open space, we used an open field setup equipped with an infrared actimeter (Panlab/Harvard Apparatus, Spain) and the ActiTrack software (SeDaCom 2.0 version pro). The experimental design consisted of a square arena 40 × 40 cm with rims 20 cm high and two square frames; an infrared detection system was used to locate the animal. The mouse was placed in the center of the arena; the animal’s behavior was monitored for 5 min. To analyze the behavioral pattern, the experimentation area was virtually divided into two zones: the central (20 × 20 cm) and the peripheral ones. The following parameters were recorded: the total distance traveled, the distance traveled in each zone, the number of rearings made (total and in each zone), the average speed of movement (cm/s), and the number of defecations and urinations, which characterize the level of “emotionality” of the animal.

#### 4.8.3. The Light–Dark Test

This experimental model designed to evaluate anxiety-related behavior is based on the earlier developed model of situational anxiety [70]. The experimental setup included the light (25 × 25 × 24 cm) and dark (19 × 11 × 12 cm) compartments connected with a partition with an opening (Panlab/Harvard Apparatus, Spain). The mouse was placed into the lit compartment with its back to the dark one, and the following parameters were automatically recorded for 10 min: the latency of the first entry into the dark compartment, the time of staying either in the light or dark compartments and the number of transitions between the compartments.

#### 4.8.4. The Acoustic Startle Reflex and Prepulse Inhibition

The setup for studying the startle reflex (Panlab/Harvard Apparatus, Spain) is able to produce any combination of sounds, noises and white noise. An experimental animal was placed into a special box and fixed there in the proper position. The level of startling was recorded by measuring the change in pressure exerted by the animal on the pad below it. The recording was performed automatically using the Packwin software (PACKWIN 2.0.) package (Panlab/Harvard Apparatus, Spain). After a 3 min period of habituation against the background of white noise (60 dB), the following signals were given:

No pulse—5 times;

Prepulse (80 dB)—5 times;

Pulse (100 dB)—5 times;

Recurrent series of prepulse/pulse signals with an interval of 60 ms—5 times.

These signals were given randomly, and the animal startles were recorded. The results were expressed as PPI calculated by the formula:

PPI = (P – P × P)/P100%,

where P is the startle response to the pulse, and PP is the startle response to the prepulse/pulse cycle.

### 4.9. Extraction of RNA from the Cell Cultures and Real-Time Polymerase Chain Reaction (RT-qPCR)

Total RNA was extracted from the mouse cerebral cortex using ExtractRNA (Evrogen, Moscow, Russia, Cat. #BC032) according to the manufacturer’s recommendations. The concentration of the extracted RNA was determined with a NanoDrop One spectrophotometer. cDNA was then synthesized using the MMLV RT reverse transcriptase kit (Evrogen, Moscow, Russia, Cat. #SK021) and random primer (Evrogen, Moscow, Russia, Cat. #SB002). Amplification was performed in qPCR mode using the qPCRmix-HS SYBR+LowROX kit (Evrogen, Moscow, Russia, Cat. #PK156L) on an AppliedBiosystems 7500 RT-PCR amplifier. The amplification process consisted of the initial holding stage 2 min 50 °C and denaturation at 95 °C for 10 min, 40 cycles of 15 s denaturation at 95 °C, 1 min annealing at 60 °C. The sequences were designed with FAST PCR 5.4 and NCBI Primer-BLAST software. Data were processed using the ΔΔCt method [71], and a control sample (total RNA from Sip1^wt/fl^-mice without BDNF overexpression, which died after pilocarpine seizures), in which the level of the target gene was taken as unity. The expression of the analyzed genes was normalized to the control Oaz1 gene encoding Ornithine decarboxylase antizyme (Oaz1).

### 4.10. Statistical Analysis

We used the Shapiro–Wilk test to determine the normality of the distribution. Statistical analyses were performed using the Student’s *t*-test, nonparametric Mann–Whitney U test or non-parametric multiple comparisons using the Kruskal–Wallis test. For the analysis of experiments with a large sample, we used two-way ANOVA, followed by Sidak’s multiple comparison test. The differences are significant (* *p* < 0.05; ** *p* < 0.01; *** *p* < 0.001; n/s—data not significant (*p* > 0.05)). MS Excel, ImageJ, Origin 2016 (OriginLab, Northampton, MA, USA) and Prism GraphPad 7 (GraphPad Software, RRID: SCR_002798) software were used for data and statistical analysis.

## 5. Conclusions

Using Ca^2+^ neuroimaging, it was possible to establish that cortical neurons obtained from heterozygous mice with deletion of the transcription factor Sip1 are characterized by increased hyperexcitability in a magnesium-free model of epileptiform activity. Increased Ca^2+^ activity of such neurons correlates with reduced expression of genes encoding BDNF, protein kinase C and PI3K with simultaneous increased expression of genes encoding excitatory glutamate receptors and key ion channels of the plasma membrane. Overexpression of BDNF in neurons of cortical cultures obtained from Sip1^wt/fl^ mice suppressed Mg^2+^-free induced epileptiform activity, which correlated with partial restoration of the expression of these genes to the level of control cells. At the organism level, BDNF overexpression in cortical neurons of Sip1^wt/fl^ mice promoted an increase in motor and exploratory activity of mice and resulted in 100% survival of these mice in the pilocarpine model of epilepsy. Such high survival of Sip1^wt/fl^ mice with BDNF overexpression correlated with an increase in the expression of genes encoding inhibitory GABA receptors, the anti-inflammatory cytokine IL-10, the hypoxia-inducible factor Hif-1α, and protective protein kinases.

## Figures and Tables

**Table 1:** Sensorimotor tests. Test for the neurological and motor functions. Statistical analyses were performed using two-way ANOVA, followed by Sidak’s multiple comparison test (Sip1^wt/fl^ group vs. Sip1^wt/fl^ + AAV-BDNF group). * *p* < 0.05; ** *p* < 0.01; *** *p* < 0.001.

		Flat Bar 3 cm	Flat Bar 2 cm	Flat Bar 1 cm	Round Bar 3 cm	Round Bar 0.5 cm
Sip1^wt/fl^	Test 1	24.5 ± 9.7	56.6 ± 21.8	62.9 ± 27.3	45.1 ± 22.4	64.5 ± 19.7
	Test 2	57.5 ± 33.8	35.0 ± 28.7	59.8 ± 3.7	29.3 ± 17.32	33.5 ± 18.6
Sip1^wt/fl^ + AAV-BDNF	Test 1	20.8 ± 8.7	25.6 ± 6.4 **	25.6 ± 11.3 **	10.2 ± 4.3 ***	18.3 ± 4.9 ***
	Test 2	5.2 ± 2.4 ***	3.8 ± 1.1 ***	3.0 ± 1.6 ***	15.2 ± 1.7 **	27.0 ± 3.9 *

**Table 2:** Startle reflex.

		Mean Max. SR Pulse	% of Negative PPI Values
Sip1^wt/fl^	Test 1	5.1 ± 3.1	37.5
	Test 2	6.5 ± 3.3	0
Sip1^wt/fl^ + AAV-BDNF	Test 1	7.6 ± 2.1	0
	Test 2	**5.7 ± 1.2**	**0**

**Table 3:** Passive avoidance test. Statistical analyses were performed using two-way ANOVA, followed by Sidak’s multiple comparison test (Sip1^wt/fl^ group vs. Sip1^wt/fl^ + AAV-BDNF group). * *p* < 0.05.

		Delta Latency	% of Animals Entering the Dark Compartment (2nd Day)
Sip1^wt/fl^	Test 1	122.1 ± 39.1	12.5
	Test 2	60.4 ± 17.7	66.6
Sip1^wt/fl^ + AAV-BDNF	Test 1	127.9 ± 58.1	12.5 ± 5
	Test 2	89.2 ± 21.2 *	60

**Table 4:** Modified Racine scale for assessing seizures in laboratory animals after pilocarpine-induced epileptiform activity [68].

Score	Behavior Stage
0	No change in behavior
1	Sudden behavioral arrest, motionless staring (with orofacial automatism)
2	Head nodding
3	Forelimb clonus with lordotic posture
4	Forelimb clonus, with rearing and falling
5	Generalized tonic–clonic activity with loss of postural tone, often resulting in death, wild jumping

## Data Availability

The data presented in this study are available on request from the corresponding author.
